# Supplementary material for: Cortex-wide spatiotemporal motifs of theta oscillations are coupled to freely moving behavior
Source: Front Syst Neurosci. 2025 Jun 19;19:1557096. doi: 10.3389/fnsys.2025.1557096 (PMC12222107; doi:10.3389/fnsys.2025.1557096)
Supplement: Supplementary Figure 1: — Cortical theta modes are robust across mice. (A) Variance explained by each mode across all sessions for each mouse, quantified through singular value decomposition computed individually for each mouse. The combined contribution of all three modes, computed individually for each mouse, was Mouse 1: 66%, Mouse 2: 63%, Mouse 3: 60%. (B) Amplitude (top row) and phase (bottom row) of Mode 1 for each mouse. (C) Amplitude (top row), phase (middle row), and vector curl (bottom row) of Mode 2 for each mouse. (D) Amplitude (top row), phase (middle row), and vector curl (bottom row) of Mode 3 for each mouse. [file Image_1.pdf]

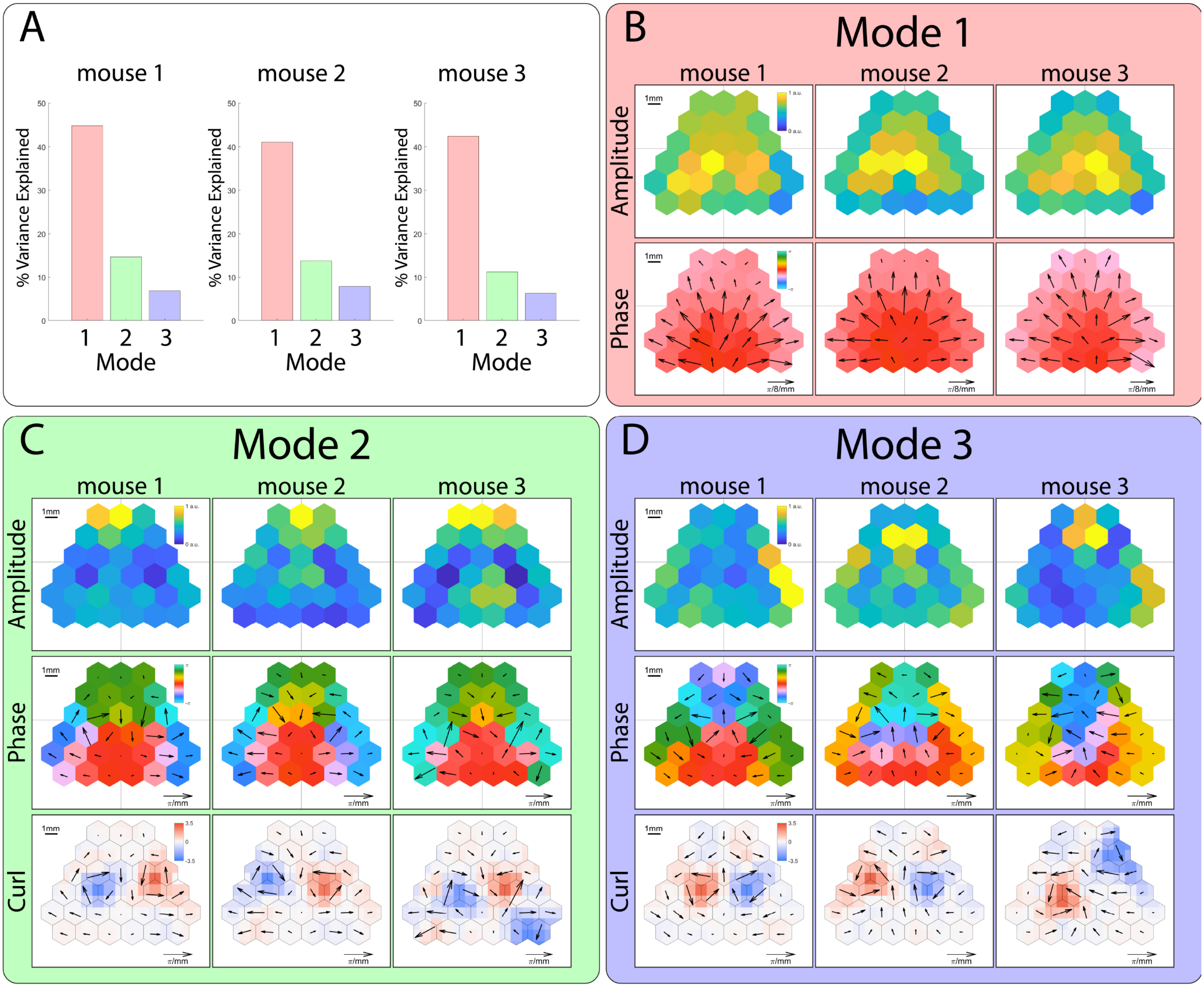

Extended Data Figure 2-1. Cortical theta modes are robust across mice.

A) Variance explained by each mode across all sessions for each mouse, quantified through singular value decomposition computed individually for each mouse. The combined contribution of all three modes, computed individually for each mouse, was Mouse 1: 66%, Mouse 2: 63%, Mouse 3: 60%.

B) Amplitude (top row) and phase (bottom row) of Mode 1 for each mouse.

C) Amplitude (top row), phase (middle row), and vector curl (bottom row) of Mode 2 for each mouse.

D) Amplitude (top row), phase (middle row), and vector curl (bottom row) of Mode 3 for each mouse.
